# Association of functional variant of aldehyde dehydrogenase 2 with acute myocardial infarction of Chinese patients

**DOI:** 10.1186/s12872-022-02738-y

**Published:** 2022-07-04

**Authors:** Qixia Jiang, Xiaoguang Li, Rukun Chen, Chuhong Wang, Xin Liu, Xingyu Wang

**Affiliations:** 1 Department of Cardiology, Tongren Hospital, Shanghai Jiao Tong University School of Medicine, Shanghai, China; 2 Department of General Surgery, Shanghai Fourth People’s Hospital Affiliated to Tongji University School of Medicine, Shanghai, China; 3 National Center for Human Genetic Resources, National Research Institute for Family Planning, Beijing, China; 4 Beijing Hypertension League Institute, Beijing, China

**Keywords:** Aldehyde dehydrogenase 2 (ALDH2), Acute Myocardial Infarction (AMI), China, Alcohol consumption, Polymorphism

## Abstract

**Background:**

The variant of ALDH2 was thought to be associated with Acute Myocardial Infarction (AMI) due to the consumption of alcohol. This study focused on how ALDH2 variant acts as an independent risk factor for AMI, regardless of alcohol consumption.

**Methods and results:**

We used the case–control INTERHEART-China study which took place at 25 centres in 17 cities in mainland China. Cases were patients with AMI and matched by age, sex, and site to controls. Information about alcohol consumption and genotype were collected. We divided cases and controls by alcohol consumption: alcohol intake group and no alcohol intake group. Then, calculated the Odd Ratio (OR) value with confidence interval (CI) at 95% level to find the association between ALDH2 variant and AMI. Results were then adjusted by sex, age, BMI, and other common risk factors of AMI. The study involves a total of 2660 controls and 2322 AMI patients. The no drink intake group showed that there was a correlation between the ALDH2 variant and AMI (OR = 1.236, 95% CI = 1.090–1.401, *p* = 0.00092). After adjustment of different risk factors this association remained (OR = 1.247, 95% CI = 1.099–1.415, *p* = 0.00062). Similar results were also obtained from the no alcohol intake group (OR = 1.196, 95% CI = 0.993–1.440, *p* = 0.05963), however, due to the limited sample size, the result was not significant enough statistically.

**Conclusion:**

From our results, ALDH2 variant is associated with the risk of AMI even in population that has no alcohol consumption. This suggests that ALDH2 variant may act as an independent risk factor for AMI.

## Introduction

According to survey in 2018, the excessive drinking rate in Chinese adults was 14.0% and 1.1% for men and women respectively and the overall harmful drinking rate was 7.1% [1]. High alcohol consumption was seen among the Chinese adult population. Alcohol intake was shown by previous studies that it has an impact on cardiovascular diseases (CVD). By increasing the level of high-density lipoprotein, moderate consumption of alcohol has been proved to have a cardiovascular protective benefit across the global population [2–7] and Chinese alone [8]. In contrast, heavy consumption of alcohol remarkably increases the risk of myocardial infarction (MI) and leads to mortality [9]. Toxic aldehydes are produced during the metabolism of alcohol in liver and could potentially damage the heart muscles and other organs as well.

The enzyme aldehyde dehydrogenase 2 (ALDH2) in alcohol metabolic pathway plays a key role in breaking down ethanol-derived acetaldehyde and endogenous lipid aldehydes [10]. ALDH2 is a member of the ALDH gene family and it is the most efficient enzyme out of the 19 human ALDH isoenzymes. ALDH2 wildtype was shown to have a protective effect on CVD as it reduced the level of harmful aldehydes through ALDH2 enzymatic activities [11, 12]. A naturally occurring single nucleotide polymorphism (SNP) rs671 of ALDH2 (at position 487 on chromosome 12, changing the amino acid from glutamate to lysine) produces less active enzymes compared to the wild-type ALDH2, which decreased the efficiency of breaking down acetaldehyde to acidic acid. The concentration of acetaldehyde greatly increases after a small amount of alcohol consumption in people with this variant. The ALDH2 variant rs671 has been seen commonly in Asian populations [13]. In China, the prevalence of ALDH2 variant differs between different regions and populations: from 9% in a Han population of certain region to 40.9% in Hakka population in South China [13, 14].

Previous studies done by Mizuno et al., Gu et al., and Jo et al. showed concordant results among Chinese, Korean and Japanese populations: ALDH2*2 alleles (ALDH2*1/*2 + ALDH2*2/*2) are more frequent in the MI patients compare to the controls and is increasing the risk of MI [15–17]. However, due to the limited sample size and insufficient collection of other AMI risk factors, past studies did not define the role of ALDH2 variant in AMI patients who do not consume alcohol. People who are homozygous or heterozygous of the ALDH2 variant tend to consume less alcohol due to the unpleasant feeling from the acetaldehyde in the system [18, 19]. Therefore, whether the ALDH2 variant rs671 acts as an independent risk factor for AMI remained to be answered.

We used INTERHEART-China study which is a multicentre case–control study to determine how ALDH2 rs671 variant is a risk factor of AMI among Chinese population with/without alcohol involvement, and sufficiently adjusted by other AMI risk factors (e.g. age, sex, BMI, alcohol intake) to minimize the cofounding effect.

## Materials and methods

### Population

The current study used INTERHEART-China study which is a case–control study of risk factors for first AMI. Methods of INTERHEART-China study were described previously [20]. AMI patients from mainland China were included in the current study. AMI is defined as: clinical symptoms and electrocardiogram showing substantial changes such as new pathological Q waves or 1 mm ST elevation in any two or more contiguous limb leads, or a new left bundle branch block or new persistent ST-T wave changes diagnostic of a non-Q wave MI, or raised concentration of troponin as defined in INTERHEART study protocol. Information was collected from a total of 2660 controls and 2322 AMI patients who had genetic samples available from 25 participating centres which distributed in 17 cities in mainland China. All controls had no history of cardiovascular diseases (CVD) at the time of the study. They were healthy visitors and non-cardiac patients from the same hospital, or people in the same community. The cases and controls were age and sex-matched. All participants provided informed consent and the current study was approved by th Ethic Committee of Beijing Hypertension League Institute.

### Parameters

Physical examinations and structured surveys were administered in cases and controls in a standard manner [21]. Information regarding age, sex, BMI, circumferences of waist and hip, smoking status, physical activities, personal and family medical history about CVD and its risk factors were collected. We defined alcohol drinkers as any individuals who had any beer, wine, liquor, or any other alcoholic beverage in the past 12 months. If yes, then participants were asked to choose if they consumed alcohol rarely (less than once a month), less than once a week, 1–2 times weekly, 3–4 times weekly, 5–6 times weekly, or daily. Alcohol intake weekly was defined as regular drinker. Participants were also asked if they had any alcohol intake within 12 months, 24 h, and 48 h prior to the onset of symptoms, diet and psychosocial stress were also assessed. Non-fasting blood samples (20 ml) were drawn and centrifuged within two hours of admission. They were then immediately frozen, stored at − 70 °C, and shipped to Beijing Hypertension League Institute for analysis.

The plasma concentrations of total cholesterol, HDLc, ApoA1, ApoB were analyzed by Hitachi 911 analyzer (Roche Diagnostics, Mannheim, Germany) and the analytic methods were described previously [11]. Total Cholesterol and HDLc were measured by standard enzymatic procedure according to manufacturer’s protocol with quality control by Assayed Human multi-sera (Randox Laboratories Ltd, UK). For ApoB and ApoA1 measurement, Immunoturbidimetric assay methods were used (Tina-quant ApoB version 2 and ApoA1 version 2 kits, Roche Diagnostics, Mannheim, Germany) with Precinorm and Precipath control samples. Interassay coefficient variation was within 5% of all lab tests.

### Genotyping

A total of 2325 cases and 2660 controls had DNA samples available. Genomic DNA was extracted with QIAGEN Blood Midi Kit (QIAGEN Germany) with manufacturer’s protocol. The genotypes of ALDH2 (rs671) were detected by TaqMan probe (ABI; C__790072_1_) with ABI-7900 real-time PCR system (Applied Biosystems, USA). A subset of 90 randomly selected samples was sequenced to validate the genotyping accuracy and yielded 100% concordance.

### Statistical analysis

T-test or appropriate non-parametric tests were used to compare continuous variables that were summarised by means and medians. The discrete variables including sex, physical activities, rs671, etc. were tested by chi-square test. Unconditional logistic regression was used for analysing the correlations between AMI and rs671. Odds ratio (OR) value was presented with a 95% confidence interval (CI). When p-value was lower than 0.05 in two-sided tests, it was considered statistically significant. The statistical analysis was carried out by SPSS 19.0.

## Results

### Baseline

2325 AMI patients from urban hospitals and 2660 age- and sex-matched controls were enrolled from 25 centres in 17 cities of mainland China. There are no significant differences in sex, waist to hip ratio, and alcohol intake between AMI patients and controls. Compare with controls, AMI patients were slightly older and had a slightly higher BMI. AMI patients also had a higher prevalence of hypertension, diabetes, current smoking, and depression compared to the controls. In lipid profiles, the AMI patients had a higher level of total cholesterol, ApoB, and ApoB/A1 ratio, and had a lower level of triglyceride, HDL, and ApoA1 (Table 1).

**Table 1:** Comparison of clinical parameters between cases and controls

	All			No alcohol intake			Alcohol intake		
	Cases	Controls	*p*	Cases	Controls	*p*	Cases	Controls	*p*
Number	2325	2660	–	1424	1662	–	901	958	–
Age, year (SD)	60.7(11.7)	59.5(11.4)	< 0.001	63.8(10.7)	61.8(10.6)	< 0.001	56.0(11.6)	55.5(11.5)	0.287
Sex, male (%)	1657(71.2)	1817(69.2)	0.132	817(57.4)	962(57.8)	0.806	840(93.0)	855(89.1)	0.003
BMI, Kg/M^2^ (SD)	24.7(3.1)	24.4(3.0)	< 0.001	24.6(3.3)	24.2(2.9)	0.004	25.0(2.9)	24.8(3.1)	0.096
Waist/hip ratio(SD)	0.88(0.08)	0.88(0.08)	0.187	0.87(0.09)	0.87(0.09)	0.528	0.90(0.07)	0.89(0.07)	0.298
SBP, mmHg(SD)	121.8(19.6)	127.9(17.2)	< 0.001	122.3(20.2)	127.8(17.6)	< 0.001	121.0(18.5)	128.0(16.5)	< 0.001
DBP, mmHg(SD)	76.2(11.8)	79.4(9.6)	< 0.001	76.1(11.8)	78.9(9.6)	< 0.001	76.3(11.8)	80.2(9.6)	< 0.001
History of hypertension (%)	884(38.0)	594(22.6)	< 0.001	576(40.5)	383(23.0)	< 0.001	308(34.1)	211(22.0)	< 0.001
Diabetes (%)	273(11.7)	81(3.1)	< 0.001	198(13.9)	53(3.2)	< 0.001	75(8.3)	28(2.9)	< 0.001
Alcohol intake (%)	903(38.8)	960(36.6)	0.108	–	–	–	–	–	–
Current smoker (%)	1013(43.6)	763(29.1)	< 0.001	434(30.5)	297(17.8)	< 0.001	579(64.3)	466(48.6)	< 0.001
Depression (%)	465(20.0)	247(9.4)	< 0.001	295(18.2)	142(8.5)	< 0.001	206(22.8)	105(10.9)	< 0.001
Total cholesterol, mmol/L(SD)	4.68(1.17)	4.58(1.14)	0.005	4.70(1.23)	4.61(1.17)	0.052	4.64(1.06)	4.51(1.08)	0.022
Triglyceride, mmol/L(SD)	1.62(1.07)	1.70(1.10)	0.021	1.61(1.03)	1.68(1.10)	0.100	1.64(1.14)	1.74(1.09)	0.089
HDL, mmol/L(SD)	1.04(0.32)	1.10(0.35)	< 0.001	1.04(0.33)	1.11(0.35)	< 0.001	1.04(0.31)	1.07(0.35)	0.089
ApoA1, g/L(SD)	1.28(0.27)	1.40(0.31)	< 0.001	1.29(0.28)	1.42(0.32)	< 0.001	1.28(0.25)	1.38(0.31)	< 0.001
ApoB, g/L(SD)	0.88(0.26)	0.83(0.23)	< 0.001	0.88(0.27)	0.84(0.23)	< 0.001	0.87(0.24)	0.83(0.22)	< 0.001
ApoB/A1(SD)	0.70(0.22)	0.61(0.20)	< 0.001	0.71(0.22)	0.61(0.20)	< 0.001	0.70(0.21)	0.62(0.22)	< 0.001

*SBP* systolic blood pressure, *DBP* diastolic blood pressure, *HDL* high-density lipoprotein, *ApoA1* apolipoprotein A1, *ApoB* apolipoprotein B

### Correlation

The ALDH2 genotype was determined in all cases and controls. Among the controls, genotype GG, GA, and AA had frequencies 70.69% (1852 of 2620), 26.41% (692 of 2620), and 2.90% (76 of 2620). G and A alleles had the frequencies of 83.89% and 16.11%. The frequencies of GG, GA, and AA alleles in AMI patients are 66.11% (1537 of 2325), 30.06% (699 of 2325), and 3.83% (89 of 2325), G and A alleles had frequencies 81.14% and 18.86%. Allele frequencies from both AMI cases and controls are in agreement with Hardy–Weinberg equilibrium (Table 2). In AMI patients, there were 788 subjects out of 2325 (33.89%) having the genotype GA or AA. Among the controls, 768 subjects out of 2620 (29.31%) were having GA or AA genotypes. Logistic regression analysis showed that there is a correlation between ALDH2 variant and AMI in a co-dominant model (OR = 1.206, 95% CI = 1.088–1.337, *p* = 0.00037). The result was consistent after adjusted by sex, age, BMI, and drinktimes (OR = 1.236, *p* = 0.00008) (Table 2). To examine the effect alcohol consumption had on AMI, cases and controls were further divided into an alcohol intake group and no alcohol intake group. Looking at the no alcohol intake group, the logistic regression showed that there was a correlation between the ALDH2 variant and AMI (OR = 1.236, 95% CI = 1.090–1.401, *p* = 0.00092). After adjusted by different risk factors (sex, age, BMI) stepwise, this association remained (OR = 1.247, 95% CI = 1.099–1.415, *p* = 0.00062) (Table 3). Our results demonstrated that ALDH2 functional variant is associated with AMI irrespective to alcohol consumption. The alcohol intake group showed a similar result after adjusted by the same risk factors (OR = 1.196, 95% CI = 0.993–1.440, *p* = 0.05963). However, due to the relatively smaller sample size for the drinking group, the result did not reach statistically significant (Table 4).

**Table 2:** The Hardy–Weinberg equilibrium in all cases and controls, and the association of ALDH2 rs671 with acute myocardial infarction subjects

ALDH2 rs671						
	Case		Control		Total	
Genotype	Number	%	Number	%	Number	%
GG	1537	66.11	1852	70.69	3389	68.53
GA	699	30.06	692	26.41	1391	28.13
AA	89	3.83	76	2.90	165	3.34
Total	2325	100	2620	100	4945	100
HWp	0.393		0.245		0.131	
G	3773	81.14	4396	83.89		
A	877	18.86	844	16.11		
Model	χ^2^	OR (95% CI)		*p*		
AA/GG	4.674	1.411 (1.031–1.931)		0.03062		
GA/GG	9.507	1.217 (1.074–1.379)		0.00205		
AA + GA/GG	11.98	1.236 (1.096–1.394)		0.00054		
AA/GA/GG	12.709	1.206 (1.088–1.337)		0.00037		
	13.239	1.212 (1.093–1.345)		0.00027	*adjusted by sex,age,bmi*	
	14.952	1.229 (1.107–1.364)		0.00011	*adjusted by sex,age,bmi,drink*	
	15.769	1.236 (1.114–1.373)		0.00008	*adjusted by sex,age,bmi,drinktimes*	
	13.995	1.219 (1.099–1.352)		0.00018	*adjusted by drink*	
	14.585	1.224 (1.104–1.359)		0.00014	*adjusted by drinktimes*	
	5.467	1.148 (1.023–1.288)		0.01938	*adjusted by sex,age,bmi,ApoB/ApoA1*	
A/G	12.994	1.211 (1.091–1.343)		0.00031

**Table 3:** The Hardy–Weinberg equilibrium in subjects with no alcohol intake among cases and controls, and the association of ALDH2 rs671 with acute myocardial infarction in no alcohol intake subjects

ALDH2 rs671						
	Case		Control		Total	
Genotype	Number	%	Number	%	Number	%
GG	881	61.87	1126	67.75	2007	65.04
GA	478	33.57	475	28.58	953	30.88
AA	65	4.56	61	3.67	126	4.08
Total	1424	100	1662	100	3086	100
HWp	0.920		0.218		0.337	
G	2240	78.65	2727	82.04		
A	608	21.35	597	17.96		
Model	χ^2^	OR (95% CI)		*p*		
AA/GG	2.841	1.362 (0.950–1.953)		0.09187		
GA/GG	10.2	1.286 (1.102–1.501)		0.00140		
AA + GA/GG	11.667	1.295 (1.116–1.502)		0.00064		
AA/GA/GG	10.973	1.236 (1.090–1.401)		0.00092		
	11.705	1.247 (1.099–1.415)		0.00062	*adjusted by sex,age,bmi*	
	5.44	1.182 (1.027–1.360)		0.01968	*adjusted by sex,age,bmi,ApoB/ApoA1*	
A/G	11.206	1.240 (1.093–1.406)		0.00082

**Table 4:** The Hardy–Weinberg equilibrium for alcohol intake group among cases and controls, and the association of ALDH2 rs671 with acute myocardial infarction in alcohol intake subjects

ALDH2 rs671						
	Case		Control		Total	
Genotype	Number	%	Number	%	Number	%
GG	656	72.81	726	75.78	1382	74.34
GA	221	24.53	217	22.65	438	23.56
AA	24	2.66	15	1.57	39	2.10
Total	901	100	958	100	1859	100
HWp	0.303		0.777		0.532	
G	1533	85.07	1669	87.11		
A	269	14.93	247	12.89		
Model	χ^2^	OR (95% CI)		*p*		
AA/GG	3.009	1.771 (0.921–3.404)		0.08278		
GA/GG	1.19	1.127 (0.909–1.397)		0.27527		
AA + GA/GG	2.154	1.169 (0.949–1.439)		0.14218		
AA/GA/GG	3.169	1.183 (0.983–1.423)		0.07507		
	3.548	1.196 (0.993–1.440)		0.05963	*adjusted by sex,age,bmi*	
	1.294	1.128 (0.917–1.387)		0.25539	*adjusted by sex,age,bmi,ApoB/ApoA1*	
A/G	3.222	1.186 (0.984–1.428)		0.07266		

In heavy drinking group (defined as having three or more drinks per week), OR is 1.336 (0.790–2.259), compare to the OR of 1.181 (0.938–1.486) for modest drinking group defined as consumption of less than three drinks per week. Further adjusting by sex, age, and BMI did not attenuate the association (Table 5).

**Table 5:** The Hardy–Weinberg equilibrium for alcohol intake group among heavy drink group and normal drink group, and the association of ALDH2 rs671 with acute myocardial infarction in alcohol intake subjects

Model	Heavy drink			Normal drink			
	χ^2^	OR (95% CI)	*p*	χ^2^	OR (95% CI)	*p*	
AA/GG	1.656	3.836 (0.425–34.635)	0.19812	2.140	1.670 (0.834–3.344)	0.14353	
GA/GG	0.624	1.243 (0.724–2.135)	0.42955	1.244	1.145 (0.903–1.451)	0.26464	
AA + GA/GG	1.172	1.336 (0.790–2.259)	0.27907	2.003	1.181 (0.938–1.486)	0.15698	
AA/GA/GG	1.689	1.373 (0.851–2.213)	0.19377	2.741	1.188 (0.969–1.456)	0.09781	
	2.068	1.423 (0.880–2.301)	0.15039	2.860	1.193 (0.972–1.464)	0.09082	*adjusted by sex,age,bmi*
	0.541	1.246 (0.993–2.241)	0.46202	1.557	1.153 (0.922–1.444)	0.21213	*adjusted by sex,age,bmi,ApoB/ApoA1*
A/G	1.815	1.399 (0.857–2.284)	0.17793	2.724	1.186 (0.969–1.453)	0.09826	

## Discussion

The current case–control study revealed that ALDH2*2 variant is significantly associated with AMI as an independent risk factor, and independent of drinking habits of individuals. The previous meta-analysis provided strong evidence that ALDH2*2 is associated with an increased risk of AMI among Chinese population [15]. Other research that focused on different populations or ethnicities also concluded that the ALDH2*2 variant is more prevalent in MI patients [15–17]. However, these studies did not take into account the drinking status of those patients. In our study, we stratified the study population into alcohol intake group and no alcohol intake group, each with case and control. We found that despite the absence of alcohol consumption, ALDH2*2 variant is still prevalent in AMI patients compared to the controls (Table 3).

Previous studies had suggested that ALDH2 has a protective effect on AMI and other diseases as it is involved in endogenous ethanol-derived aldehydes metabolism such as 4-hydroxy-2-nonenal (4-HNE) [12, 22]. The lacking of the functional ALDH2*1 allele causes the deficiency of the metabolism. People with this deficiency who frequently consume alcohol will be more like to experience CVD due to the toxicity from the accumulated acetaldehyde [15, 19, 23]. Our results are consistence with the findings that ALDH2 wildtype is protective, while ALDH2*2 variant is a risk factor for AMI which is independent of the alcohol consumption of individuals. Comparing the no alcohol intake group and the alcohol intake group, there was no significant difference in the OR calculated after adjusted other covariates such as sex, age, and BMI. The sensitivity assay we performed showed that the correlation between AMI and ALDH2 variant persist when we remove individuals with alcohol intake. It is reasonable to conclude that ALDH2 variant is the risk factor for AMI independent of alcohol intake.

To our knowledge, this is the first time to report the independent correlation between ALDH2 variant and AMI in people with and without alcohol consumption. Although the statistical analysis showed a correlation between individuals carrying ALDH2*2 allele had higher risk of AMI, the underlying mechanism remains unclear. ALDH2 enzyme plays an important role in alcohol metabolism as well as endogenous aldehyde detoxifications as it catalyses the degradation of aldehydes and derivatives. Activation of ALDH2 can enhance aldehyde detoxification, hence reduce the damage to the heart muscles and lower the risk of AMI [16]. As the variant of ALDH2 produces dysfunctional proteins, detoxification becomes less efficient. Toxic aldehydes are building up and damage the heart muscles.

It has been shown that in animal studies, wildtype ALDH2 reduced ethanol-induced elevation in cardiac acetaldehyde levels. AMI ethanol challenge deteriorated myocardial and cardiomyocyte contractile function [12]. Another study using ALDH2/LDLR knockout mice to illustrate the new pathway of ALDH2 to maintain lysosomal function, autophagy, and degradation of oxidized low-density lipid protein. ALDH2*2 could attenuate and interrupt ALDH2 function, resulting in increased foam cells due to impaired lysosomal function [24]. It is reasonable to speculate that in human, ALDH2*2 with reduced enzymatic function will promote the formation of atherosclerosis, which is the underlying cause of acute myocardial infarction. This results also explains why ALDH2*2 is an independent risk factor even without alcohol involvement. Further prospective studies with Mendalian randomization studies are required to verify the findings.

The strength of this study is that it used a large sample size which came from multiple representative regions in mainland China. All participants were evaluated with the same standard across all centres. All cases and age sex-matched controls were recruited from the same community or hospitals, which limited the impacts of lifestyles and behavioural interventions had on the study and reduced the inherent selection bias in case–control studies. Also, as data around risk factors for AMI was carefully documented in the INTERHEART-China study, we were able to adjust the association by known AMI risk factors. This allowed us to better evaluate the genetic variant as the independent variable and produce accurate estimates of risk assessment. At the same time, we do note the limitation of our study. The cases and controls involved in this study may answered the questions selectively, especially questions around alcohol consumption and smoking status. This makes the study vulnerable to recall and may leads to social desirability bias [25]. As the samples were recruited from AMI patients and controls in their community, only a small proportion of cases and controls were alcohol drinkers. This resulted in that our statistical analysis of the alcohol intake group was underpowered, even direction and strength of association are consistent, a larger sample size is needed to confirm the result.

In conclusion, ALDH2 rs671 variant is associated with AMI risk, independent to the alcohol involvement. The result supports that ALDH2*2 allele is an independent risk factor of AMI.

## Data Availability

The datasets generated and/or analysed during the current study are available in the National Genomics Data Center repository, https://ngdc.cncb.ac.cn (PRJCA008592).

## Abbreviations

CVD: Cardiovascular Diseases
AMI: Acute Myocardial Infarction
ALDH2: Aldehyde Dehydrogenase 2
SNP: Single Nucleotide Polymorphism
